# Diphlorethohydroxycarmalol Isolated from *Ishige okamurae* Exerts Vasodilatory Effects via Calcium Signaling and PI3K/Akt/eNOS Pathway

**DOI:** 10.3390/ijms22041610

**Published:** 2021-02-05

**Authors:** Yu An Lu, Yunfei Jiang, Hye-Won Yang, Jin Hwang, You-Jin Jeon, Bomi Ryu

**Affiliations:** 1 Department of Marine Life Science, Jeju National University, Jeju 63243, Korea; annie.lu1213@gmail.com (Y.A.L.); jiangyunfei0310@aliyun.com (Y.J.); koty221@naver.com (H.-W.Y.); ghkdwls9280@naver.com (J.H.); 2 Marine Science Institute, Jeju National University, Jeju 63333, Korea

**Keywords:** Diphlorethohydroxycarmalol, Calcium, VEGFR2, Acetylcholine receptor, NO production, Vasodilation

## Abstract

Nitric oxide (NO) is released by endothelial cells in the blood vessel wall to enhance vasodilation. Marine polyphenols are known to have protective effects against vascular dysfunction and hypertension. The present study is the first to investigate how diphlorethohydroxycarmalol (DPHC) isolated from *Ishige okamurae* affects calcium levels, resulting in enhanced vasodilation. We examined calcium modulation with the well-known receptors, acetylcholine receptor (AchR) and vascular endothelial growth factor 2 (VEGFR2), which are related to NO formation, and further confirmed the vasodilatory effect of DPHC. We confirmed that DPHC stimulated NO production by increasing calcium levels and endothelial nitric oxide synthase (eNOS) expression. DPHC affected AchR and VEGFR2 expression, thereby influencing transient calcium intake. Specific antagonists, atropine and SU5416, were used to verify our findings. Furthermore, based on the results of in vivo experiments, we treated *Tg(flk:EGFP)* transgenic zebrafish with DPHC to confirm its vasodilatory effect. In conclusion, the present study showed that DPHC modulated calcium transit through AchR and VEGFR2, increasing endothelial-dependent NO production. Thus, DPHC, a natural marine component, can efficiently ameliorate cardiovascular diseases by improving vascular function.

## 1. Introduction

Hypertension is a medical condition characterized by persistently high pressure on the blood vessels [1]. The higher the pressure, the more the heart has to pump to overcome the resistance from blood vessels due to the blood pressure [2]. Therefore, vasodilation plays a vital role in preventing hypertension. Nitric oxide (NO) release by endothelial cells in the blood vessel wall has been reported to improve vascular tone [3]. Thus, it is now widely accepted that NO, along with the endothelium-derived relaxing factor [4], exerts vasodilatory and antiproliferative effects to maintain vascular homeostasis. NO is synthesized by the endothelial nitric oxide synthase (eNOS), which is activated by blood shear-stress and agonists such as acetylcholine [5].

Additionally, NO production by eNOS activation is mediated by the release of calcium ions from subsarcolemmal storage sites through two distinct mechanisms: (i) shearing stress on the vascular endothelium generated by the blood flow induces calcium release and the subsequent eNOS activation associated with the phosphoinositide 3-kinase (PI3K)/protein kinase B (Akt)-dependent signaling pathway [6]; (ii) endothelial receptors recognizing the vascular endothelial growth factor (VEGF) or acetylcholine (Ach) stimulate calcium release and subsequent NO production [5,7]. Conversely, endothelium dysfunction causing reduced intracellular [Ca^2+^] concentration and eNOS-derived NO levels can contribute to hypertension [8]. Therefore, NO homeostasis by endothelial cell regulation is critical for modulating the vascular tone [9]. Thus, the investigation of NO-based therapeutics is important in hypertension prevention.

Marine algae have been widely used as medicinal foods that harbor several vascular health-improving properties, such as antioxidative, anti-inflammatory, and antithrombotic properties, as well as the ability to modulate NO bioavailability [10]. *Ishige okamurae* (IO) is an edible brown alga widely distributed throughout the temperate coastal zone of East Asia [11]. Several studies have suggested the beneficial effect of IO in ameliorating cardiovascular conditions by inhibition of angiotensin I-converting enzyme (ACE) and maintenance of vascular homeostasis in vivo and in vitro [12]. Furthermore, diphlorethohydroxycarmalol (DPHC) is a major phenolic compound isolated from IO [11]. The strong ability of DPHC to regulate vascular endothelial cells and improve cardiovascular disease has been previously reported [13]. However, the underlying molecular mechanisms involved in DPHC-mediated vasodilation in endothelial cells remain unknown. Hence, the present study aimed to evaluate the effects of DPHC on calcium levels and NO production, and consequently, vasodilation.

## 2. Results

### 2.1. Cytotoxicity of DPHC and Time-Dependent NO Production in EA.hy926 Cells

**Figure 1:**
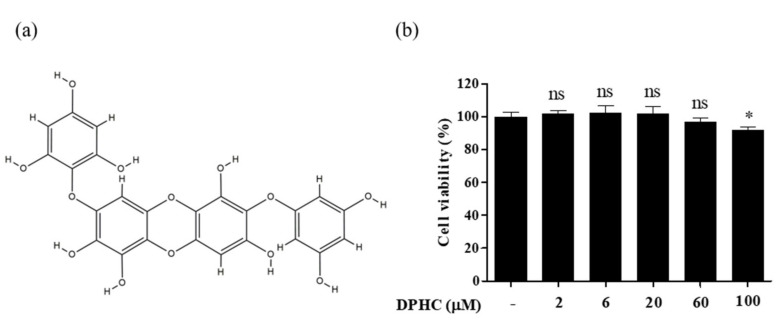
(**a**) Structure of diphlorethohydroxycarmalol (DPHC) (**b**) Cell viability analysis of EA.hy926 cells treated with different concentrations of DPHC. EA.hy926 cells were incubated with different concentrations of DPHC (0, 6, 20, 60, and 100 μM) for 24 h, and cell viability was determined by 3-(4-5-dimethyl-2yl)-2-5-diphynyltetrasolium bromide (MTT) assay. Each column and bar represent the mean ± standard deviation (S.D.). * *p* < 0.05, significant difference compared to the control group. DPHC: diphlorethohydroxycarmalol; ns: not significant.

DPHC (Figure 1a), a phlorotannin isolated from IO extract, constitutes 2.2% ± 0.43 of IO (Supplementary Materials). DPHC cytotoxicity in EA.hy926 cells is shown in Figure 1b. We found that DPHC was not toxic when used at concentrations of 6, 20, and 60 μM, but upon treatment with 100 μM DPHC, the cell viability was slightly decreased. Based on our previous study, we performed subsequent experiments with only non-toxic DPHC concentrations.

**Figure 2:**
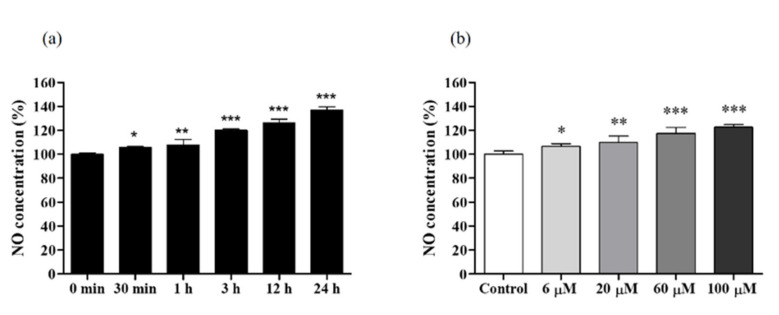
NO production in EA.hy926 cells treated with 60 μM of diphlorethohydroxycarmalol (DPHC). (**a**) Levels of time-dependent NO production in EA.hy926 cells treated with 60 μM of DPHC. (**b**) NO production in EA.hy926 cells induced by different concentrations of DPHC. Experiments were performed in triplicates. Each column and bar represent the mean ± standard deviation (S.D.). * *p* < 0.05, ** *p* < 0.01. *** *p* < 0.001, significant difference compared to the control group.

NO production is dynamically correlated with concentrations of the stimulatory agent and the incubation time. Hence, we used the highest non-toxic concentration of DPHC (60 μM) to investigate the optimal time for NO formation in EA.hy926 cells. We measured intracellular NO levels at five different time points (30 min, 1 h, 3 h, 12 h, and 24 h). NO production levels were significantly increased starting from 30 min, and the peak was observed at 24 h (Figure 2a). Therefore, a 24-h incubation period was selected as the optimal incubation time for further experiments. Next, we investigated the levels of dose-dependent NO production in EA.hy926 cells treated with different concentrations of DPHC (6, 20, 60, and 100 μM). We observed that DPHC induced NO production in a dose-dependent pattern (Figure 2b). Of all the concentrations tested, the highest NO production was observed at 60 and 100 μM of DPHC treatment.

### 2.2. DPHC Promoted Phosphorylation in the PI3K/Akt/eNOS Pathway in EA.hy926 Cells

**Figure 3:**
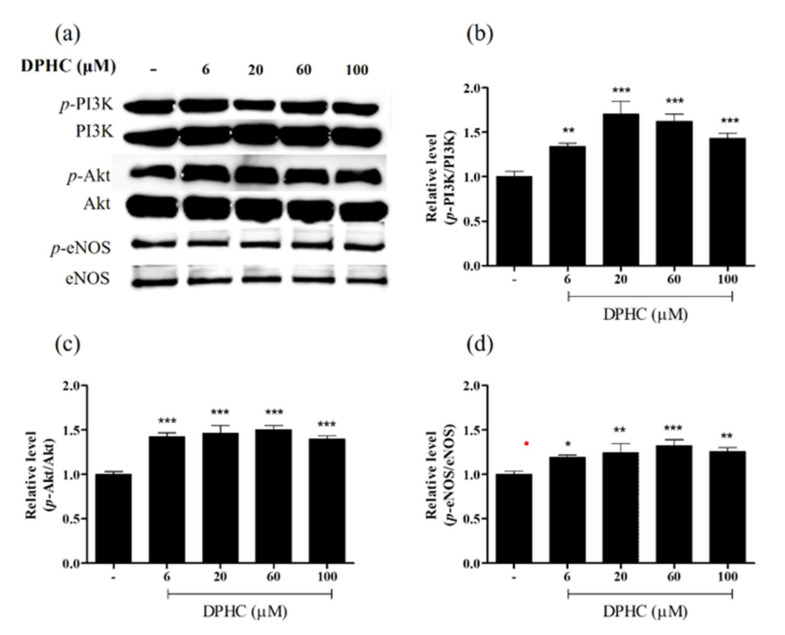
Evaluation of the expression levels of vasodilation-associated proteins. (**a**) Representative Western blot analysis. (**b**,**c**) Quantification of phosphorylated (p)-PI3K/PI3K (**b**), p-Akt/Akt (**c**), and p-eNOS/eNOS (**d**) in EA.hy926 cells treated with different concentrations of DPHC. The protein bands were ultimately developed and photographed with the FUSION Solo Vilber Lourmat system. Quantitative data were analyzed using Image J 1.50i software (NIH, USA). Results are expressed as the mean ± standard deviation (S.D.) of three independent experiments. * *p* < 0.05, ** *p* < 0.01. *** *p* < 0.001, significant difference compared to the control group. DPHC: diphlorethohydroxycarmalol; PI3K: phosphoinositide 3-kinase; Akt: protein kinase B; eNOS: endothelial nitric oxide synthase.

Since phosphorylation of PI3K and Akt promotes eNOS activity and further enhances NO production [14], we evaluated whether the enhancement of NO production was related to this pathway; protein expression levels of *p*-PI3K, *p*-Akt, and *p*-eNOS were examined by Western blotting of EA.hy926 cells treated with different concentrations of DPHC. The relative levels of *p*-PI3K showed a dose-dependent increment (Figure 3b). However, the pattern showed a slight decrease from 60 to 100 μM. After checking the relative levels by statistical analyses, there was a non-significant difference between these two concentrations (data not shown). In addition, compared to the control group, the DPHC treatments remarkably enhanced *p*-Akt expression (Figure 3c). The expression of *p*-eNOS showed a significant increment at 6, 20, and 60 μM of DPHC compared to control (Figure 3d). Nevertheless, the expression of *p*-PI3K, *p*-Akt, and *p*-eNOS at 100 μM of DPHC treatment showed a slightly decreased trend; the low cell viability was considered the main factor that affected the protein expression. Therefore, to eliminate the influence of cell viability, the 100 μM concentration was excluded from further analyses. Based on these results, we hypothesized that DPHC might activate eNOS through the PI3K/Akt/eNOS signaling pathway.

### 2.3. Regulatory Effects of DPHC on the [Ca^2+^]_cytol_ and the [Ca^2+^]_ER_ Levels

**Figure 4:**
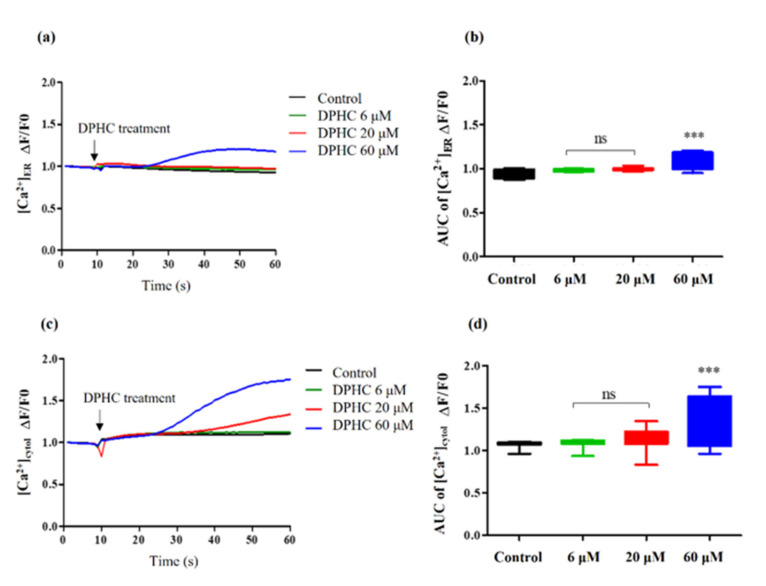
Quantification of the [Ca^2+^]_ER_ and [Ca^2+^]_cytol_ levels stimulated by different concentrations of DPHC in EA.hy926 cells. (**a**,**b**) The traces (**a**) and box plots (**b**) indicating the levels of [Ca^2+^]_ER_. (**c**,**d**) The traces (**c**) and box plots (**d**) indicating the levels of [Ca^2+^]_cytol_. For statistical significance, each sample treatment group was compared to the control group. Experiments were performed in triplicates. * *p* < 0.05, ** *p* < 0.01, and *** *p* < 0.001. ns: not significant; AUC: area under the curve; DPHC: diphlorethohydroxycarmalol; [Ca^2+^]_ER_: calcium level in the endoplasmic reticulum; [Ca^2+^]_cytol_: calcium level in the cytosol; PSS: physiological salt solution.

In the present study, the [Ca^2+^]_cytol_ level represented the calcium levels in the cytosol, which can be influenced by the activation of specific receptors such as vascular endothelial growth factor 2 (VEGFR2) and acetylcholine receptors (AchR). The [Ca^2+^]_ER_ level represented calcium stored in the endoplasmic reticulum (ER). To investigate whether DPHC could upregulate [Ca^2+^] levels in endothelial cells, [Ca^2+^]_ER_ and [Ca^2+^]_cytol_ levels were measured separately. After treating the cells with 60 μM of DPHC, [Ca^2+^]_ER_ levels were elevated after 20 s (Figure 4a). We quantified the increase in [Ca^2+^]_ER_ levels by measuring the area under the curve (AUC). We found that 60 μM of DPHC significantly raised [Ca^2+^]_ER_ levels in EA.hy926 cells (Figure 4b, *p* < 0.001). Moreover, [Ca^2+^]_cytol_ levels were dramatically increased starting from 30 s after the treatment, with a peak detected around 60 s. At 60 s, [Ca^2+^]_cytol_ levels were 1.75 times higher than that in the untreated control (Figure 4c). Quantification of [Ca^2+^]_cytol_ levels revealed that 60 μM of DPHC significantly promoted an increase in the [Ca^2+^]_cytol_ levels, compared to other DPHC concentrations (Figure 4d, *p* < 0.001).

### 2.4. DPHC Modulated [Ca^2+^] Levels by Activating AchR and VEGFR2

**Figure 5:**
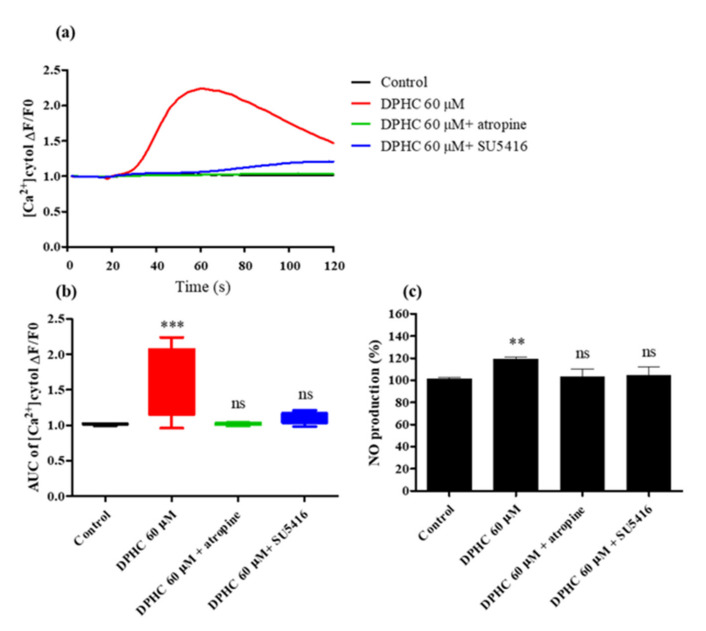
Influence of specific antagonists on [Ca2+]_cytol_ levels in EA.hy926 cells treated with DPHC. (**a**,**b**) Traces (**a**) and box plots (**b**) indicating [Ca2+]_cytol_ levels in response to treatment with DPHC (60 μM) and antagonists. (**c**) Effect of DPHC on NO production in EA.hy926 cells pretreated with atropine or SU5416. The NO levels were detected by adding 10 μM of 4 amino-5-methylamino-2′, 7′-difluorescein diacetate (DAF-FM DA). Experiments were performed in triplicates. Each column and bar represent the mean ± standard deviation (S.D.). ** *p* < 0.01. *** *p* < 0.001, significant difference compared to the control group. ns: not significant; AUC: area under the curve; DPHC: diphlorethohydroxycarmalol; [Ca2+]_cytol_: calcium level in the cytosol.

[Ca^2+^] levels can be influenced by the activation of VEGFR2 and AchR [15,16]. Thus, to investigate whether DPHC-induced NO formation was related to AchR and VEGFR2 activation, the respective specific antagonists, atropine and SU5416, were used. We tested different concentrations of atropine and SU5416 to attain the optimal inhibition conditions. Accordingly, a 2-h incubation with 60 μM of atropine and 100 μM of SU5416 showed the strongest inhibitory activity when administered separately. Therefore, the above concentrations were used in subsequent experiments. An increase in [Ca^2+^]_cytol_ levels was only observed in the DPHC treatment group, with a maximal calcium concentration observed at about 30 s after treatment, followed by a gradual decline. As expected, [Ca^2+^]_cytol_ levels were remarkably decreased when cells were treated with atropine and SU5416 compared to cells treated with DPHC. Additionally, there was no significant difference in [Ca^2+^]_cytol_ levels between the antagonist-treated and control groups (Figure 5a,b, *p* < 0.001). Thus, these findings indicate that the modulation of [Ca^2+^] levels by DPHC was closely related to AchR and VEGFR2 activation.

Having found that [Ca^2+^] levels could be regulated by AchR and VEGFR2 activation, we further hypothesized that [Ca^2+^] levels would deeply influence NO production. To prove our hypothesis, cells were pretreated with atropine or SU5416 and incubated for 2 h, followed by the addition of 60 μΜ of DPHC. After 24 h, intracellular NO concentrations were measured using 4 amino-5-methylamino-2′, 7′-difluorescein diacetate (DAF-FM DA). We observed that both antagonists significantly suppressed NO formation upon DPHC stimulation (Figure 5c). Taken together, our results showed that the modulation of [Ca^2+^]_cytol_ levels by AchR and VEGFR2 activation was sufficient and necessary for NO formation.

### 2.5. DPHC Enhanced Vasodilation in the Tg(flk:EGFP) Transgenic Zebrafish

**Figure 6:**
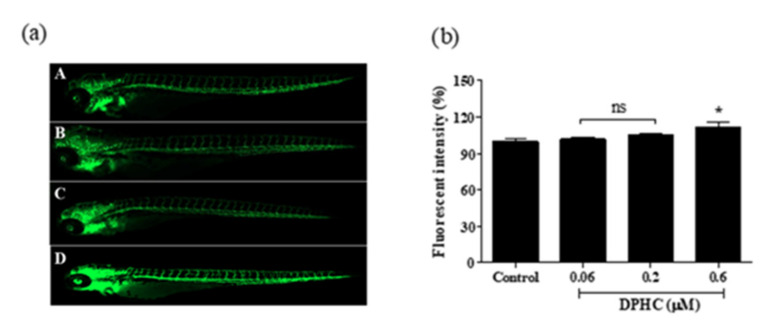
DPHC induces vasodilation in the whole-body vasculature in a Tg(flk:EGFP) transgenic zebrafish model. (**a**) Representative images of the Tg(flk:EGFP) transgenic zebrafish larva’s whole body captured using a fluorescence microscope (4×). A: 0 μM of DPHC (Control); B: 0.06 μM of DPHC; C: 0.2 μM of DPHC; D: 0.6 μM of DPHC. (**b**) Quantification of the whole-body fluorescence intensity. Each column and bar represent the mean ± standard deviation (S.D.), *n* = 8 per group. * *p* < 0.05, significant difference compared to the control group. ns: not significant; DPHC: diphlorethohydroxycarmalol.

Due to some characteristics, such as rapid development, ease of genetic manipulation, advanced genomic resources, as well as similar organ systems and gene functions as humans, the zebrafish (*Danio rerio*) provides significant advantages in terms of our understanding of cardiovascular disease causalities. Particularly, the embryos develop rapidly, exhibiting optical transparency during the first-hour post-fertilization, allowing for direct observation using light microscopy. These features contribute to the emergence of the zebrafish animal model as a useful and valuable tool for cardiovascular research [17,18]. To investigate whether DPHC could exert a vasodilation effect *in vivo*, we used a *Tg(flk:eGFP*) transgenic zebrafish model, in which the vascular endothelial cells are fluorescently stained with the enhanced green fluorescent protein (eGFP). After the treatment of samples, changes in the vessel diameter and the fluorescence intensity can be observed easily using confocal microscopy (Figure 6a). Zebrafish larvae were treated with different concentrations of DPHC (0, 0.06, 0.2, and 0.6 μM) from 3 days post fertilization (dpf) to 7 dpf. Treatment with 0.6 μM of DPHC significantly increased the fluorescence intensity in the whole body compared to control larvae (Figure 6b). However, we did not observe any difference between the 0.06 and 0.2 μM DPHC treatments.

**Figure 7:**
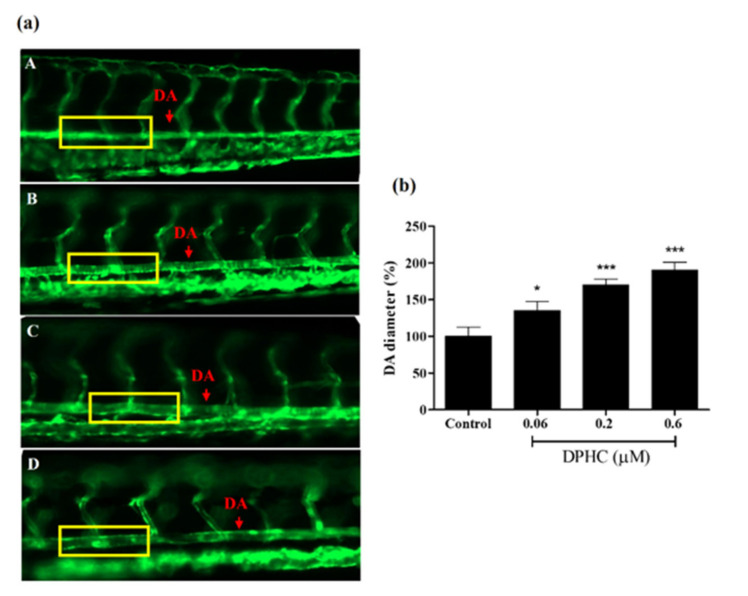
Vasodilation observed by changes in the vessel diameter in a Tg(flk:EGFP) transgenic zebrafish model. (**a**) Images of the vessel were captured using a fluorescence microscope (20×). (A–D) Vasodilation observed by treatment with DPHC at 6 dpf. A: 0 μM of DPHC (Control); B: 0.06 μM of DPHC; C: 0.2 μM of DPHC; D: 0.6 μM of DPHC. (**b**) Measurement of the DA diameter. Each column and bar represents the mean ± standard deviation (S.D.), *n* = 8 per group. * *p* < 0.05, *** *p* < 0.001, significant difference compared to the control group. DPHC: diphlorethohydroxycarmalol; DA: dorsal aorta.

Additionally, the dorsal aorta (DA) is the major trunk axial artery and is one of the first vessels to assemble in the early developmental stages in all vertebrates. A previous study demonstrated that the DA of the zebrafish acquires a vascular smooth muscle cell-containing vascular wall similar to that found in other vertebrates. Therefore, evaluation of the DA diameter can be considered direct evidence for vasodilation. As shown in Figure 7, the DA diameter significantly increased with DPHC treatment (0.06, 0.2, and 0.6 μM) compared to the control group. Therefore, our findings proved that DPHC could be a potential vasodilator.

## 3. Discussion

Several polyphenols extracted from terrestrial plants have been reported for their antihypertensive effects caused by increasing NO bioavailability and alleviation of vasoconstriction [19,20]. Marine algae are also a potentially rich resource of substances with beneficial health effects, but the molecular mechanisms underlying their effect on blood pressure are seldom known. Previous in vitro studies have reported that marine polyphenols isolated from *Ecklonia Cava,* such as phlorofucofuroeckol A, dieckol, and eckol, exert vasodilatory effects by increasing NO production and eNOS expression [21,22]. Furthermore, these polyphenols exhibited antihypertensive effects by decreasing the systolic blood pressure in hypertension mouse models [23]. In contrast, only protective effects against cardiovascular diseases, such as inhibition of diabetic disorders and obesity inhibition in 3T3-L1 adipocytes, have been reported for IO and DPHC [24]. The vasodilatory or antihypertensive properties of IO or DPHC were rarely mentioned. Therefore, we aimed to investigate the molecular mechanisms of vasodilation by DPHC using in vitro and in vivo models.

A concentration-dependent increase in NO production was observed in the DPHC treatment groups. It has been reported that multiple mechanisms control NO production via eNOS activation. First, the PI3K pathway members, including its downstream molecule, Akt, are essential regulators; activated Akt directly phosphorylates ser1177 on eNOS, enhancing the binding activity of [Ca^2+^]/calmodulin [25]. Based on these aspects, we systematically examined protein expression in the PI3K/Akt/eNOS axis and measured calcium transit. Indeed, dose-dependent increments in PI3K, Akt, and eNOS were observed under DPHC treatment. Thus, we confirmed that DPHC-induced activation of the PI3K/Akt/eNOS pathway is essential in promoting NO formation in endothelial cells. Furthermore, activated eNOS promotes the binding of calcium ions to calmodulin, a multifunctional intermediate calcium-binding messenger protein. Once [Ca^2+^] is bound to calmodulin, the [Ca^2+^] signal transduction pathway is activated [26]. As such, calcium transit is also considered critical in NO generation [27]. Kida et al. indicated that increasing the [Ca^2+^]_ER_ level would activate the calmodulin-bound domain of eNOS, resulting in NO production [28]. Additionally, higher [Ca^2+^]_cytol_ levels led to NO formation, which was shown in human umbilical vein endothelial cells [29]. In this study, we investigated the role of [Ca^2+^] signaling under DPHC administration. After adding 60 μM of DPHC, it was possible to detect a remarkable increase in [Ca^2+^]_cytol_ and [Ca^2+^]_ER_ levels after 30 s, characterized by a 60 s oscillation peak phase. This increase was completely prevented by different antagonists (VEGFR2 and AchR antagonists). Theoretically, after the peak phase, a stabilization declining phase, also characterized by the presence of less intense and less frequent oscillations, would follow [30]. Nevertheless, within 60 s, we did not observe any decreasing trend in [Ca^2+^]_cytol_ and [Ca^2+^]_ER_ levels (Figure 4a,c).

When the cells are stimulated, such as during membrane depolarization, extracellular signaling molecules or intracellular messengers enhance [Ca^2+^]_cytol_ levels. This increment results from either an influx of [Ca^2+^] from the outside of the cell via ion channels on the plasma membrane or the release of [Ca^2+^] from internal stores (such as the ER). The increase in [Ca^2+^]_cytol_ level is precipitous and is followed by a decline in [Ca^2+^] levels, maintained by the homeostatic regulation of [Ca^2+^] concentration [31]. Accordingly, having found that DPHC can effectively raise [Ca^2+^]_cytol_ and [Ca^2+^]_ER_ levels, we further focused on investigating how DPHC affects calcium regulation via cell membrane receptors (VEGFR2 and AchR). The muscarinic AchR family is divided into 5 subtypes: M1, M2, M3, M4, and M5 [32]. The activation of M3 receptors in vascular endothelial cells induces potent vasodilatation, and this process occurs via the release of an endothelium-derived relaxing factor, such as NO [33]. Ren et al. showed that acetylcholine induces vasodilatation by activating M3 receptors on endothelial and smooth muscle cells [34]. Moreover, AchR activation can induce a downstream pathway involved in the conversion of phosphatidylinositol biphosphate (PIP_2_) into the two secondary messengers, inositol-1,4,5-trisphosphate (IP_3_) and diacylglycerol (DAG), by phospholipase-C (PLC). IP_3_ diffuses into the cytosol and binds to its receptor (IP3R) on the ER, triggering the release of Ca^2+^ ions [35]. The rise of free Ca^2+^ ions mediates activation of NO generation via the phosphorylation of proteins, such as Akt and eNOS [36]. As we expected, the pretreatment of cells with atropine, a specific antagonist of AchR, completely blocked DPHC-induced rise in [Ca^2+^]_cytol_. In addition, we observed that the level of NO was obviously suppressed by atropine. Therefore, we concluded that DPHC-induced rise in [Ca^2+^]_cytol_ and NO formation could proceed with AchR activation in EA.hy926 cells. Another potential regulator of vasodilation is VEGFR2, mainly expressed in the vascular endothelium [37]. When VEGF binds to VEGFR2, the receptor undergoes dimerization and phosphorylation of its tyrosine residues, triggering a downstream phosphorylation cascade targeting pro-angiogenic mediators [38]. Among the activated mediators are the PLCγ1/calcium and PI3K/Akt/eNOS pathways [39]. Both pathways are associated with eNOS activation and NO synthesis. VEGFR2 data (Figure 5) demonstrated a similar trend with the AchR results. Treatment with 60 μM of DPHC could not raise [Ca^2+^]_cytol_ levels or NO production in EA.hy926 cells pretreated with SU5416. Therefore, we hypothesize that DPHC triggered an increase in [Ca^2+^]_cytol_ levels by regulating AchR and VEGFR2 activation, resulting in NO production.

Based on the in vitro findings, we further examined the vasodilatory effect of DPHC in a transgenic zebrafish model. Compared to the mouse, zebrafish is a more economically feasible model for in vivo screening of small-molecule compounds in terms of cost-quality. Because the vascular structure is easily visualized, numerous studies on vascular-related disorders have been performed in different types of transgenic zebrafish models, such as angiogenesis [40], vasoconstriction [41], and diabetes-induced endothelial dysfunction models [42]. The *Tg(flk:eGFP)* transgenic zebrafish model is one of the most common strains for studying vessel diameter change. Once the vessel diameter is increased by sample treatment, a higher percentage of fluorescence intensity can be observed [12]. Therefore, the *Tg(flk:eGFP)* strain of zebrafish was employed in our in vivo experiments. As expected, treatment with 0.6 μM of DPHC significantly increased the fluorescence intensity in the whole body of zebrafish larvae (Figure 6). In other words, data indicated that treatment with 0.6 μM of DPHC could effectively promote vasodilation. Further, we demonstrated clear evidence of vessel diameter increment in DA vessels. As previously stated, the DA is directly connected to the heart; thus, the DA diameter is important in the modulation of heartbeats and blood circulation [43,44]. Our results provide proof that treatment with DPHC could dramatically enhance vasodilation in a dose-dependent manner in in vivo models (Figure 7). However, due to the limited equipment, other cardiovascular parameters, such as blood flow velocity and cardiac output, need to be confirmed by further experiments.

In summary, we demonstrated that DPHC, a natural compound, can be a potential endothelium-dependent vasodilator that acts by modulating calcium transit through AchR and VEGFR2, increasing endothelial-dependent NO production and resulting in vasodilation in the *Tg(flk:eGFP)* transgenic zebrafish model.

## 4. Materials and Methods

### 4.1. Reagents

Dulbecco’s modified Eagle’s medium (DMEM) and penicillin/streptomycin solution were purchased from GIBCO (Grand Island, NY, USA). Fetal bovine serum (FBS) was obtained from Merck (Sacramento, CA, USA); dimethyl sulfoxide (DMSO) and 3-(4-5-dimethyl-2yl)-2-5-diphynyltetrasolium bromide (MTT) were purchased from Sigma-Aldrich (St. Louis, MO, USA). The intracellular NO production was detected using DAF-FM DA (Thermo Fisher Scientific, Waltham, MA, USA); the total NO production was measured using the Griess assay (Promega Corporation, Madison, WI, USA). Calcium levels were quantified using Fluo-4-AM dye (1-[2-amino-5-(2,7-difluoro-6-hydroxy-3-oxo-9-xanthenyl) phenoxyl]-2-(2-amino-5-methylphenoxy) ethane-N, N, N′, N′-tetraacetic acid, pentaacetoxymethyl ester) (Thermo Fisher Scientific, Waltham, MA, USA ). Atropine, a specific AchR antagonist, was purchased from Sigma Aldrich. SU5416, a VEGFR2 inhibitor, was obtained from Tocris Bioscience (Bristol, UK).

### 4.2. DPHC Isolation

DPHC isolation was performed as previously described [13]. In brief, IO leaves were collected from Jeju Island, South Korea. IO specimens were washed with running water to remove salt, sand, and epiphytes attached to the surface. Next, IO specimens were lyophilized and ground to obtain a dry powder. Dried IO powder was extracted in 50% ethanol under refluxing conditions. The extract was concentrated and freeze-dried (final yield: 87 g). Centrifugal partition chromatography (CPC) (CPC240, Tokyo, Japan) was performed with a portion of the extract. The CPC solvent system was composed of a mixture of *n*-hexane, EtOAc, MeOH, and H_2_O. Further purification was performed using an HPLC system (Milford, Massachusetts, USA) equipped with a YMC-Pack ODS-A column (YMC Co., Ltd., Kyoto, Japan). The sample was eluted using an isocratic solvent system.

### 4.3. Cell Culture and Cell Viability Analysis

Human cardiovascular endothelial cell line EA.hy926 was acquired from the American Type Culture Collection (ATCC; Manassas, VA, USA) and cultured in DMEM supplemented with 100 U/mL penicillin, 0.1 mg/mL streptomycin, and 10% FBS. Cells were grown in a humidified incubator at 37 °C, in an atmosphere with 5% (*v*/*v*) CO_2_. Cells at 2–5 passages were used for all experiments.

EA.hy926 cell viability was assessed by an MTT assay. A total of 1 × 10^5^ cells/well (180 μL of cell suspension) were seeded in a 96-well plate and incubated at 37 °C for 24 h. Cells were treated with different concentrations (6, 20, and 60 μM) of DPHC for another 24 h. Then, 100 μL (concentration: 2 mg/mL) of MTT was added to the wells, and cells were incubated for 2 h. After 2 h, the medium was replaced with 150 μL of DMSO. The supernatant was collected, and the absorbance was measured using a microplate reader at 540 nm (Synergy HT, BioTek Instruments, Winooski, VT, USA).

### 4.4. Quantification of the Intracellular NO Production

DAF-FM DA is a fluorescent probe used to detect intracellular NO. DAF-FM DA is a cell-permeable deacetylated form of DAF-FM and is hydrolyzed by intracellular esterases to form a cell-impermeable DAF-FM that reacts with NO. Therefore, the fluorescence intensity in cells was used to evaluate intracellular NO levels [45]. EA.hy926 cells were pre-incubated with 10 μM of DAF-FM DA reagent for 30 min in the dark. The mean fluorescence intensity was then measured using a spectrofluorometer (Synergy HT, BioTek Instruments, Italy).

### 4.5. Quantification of the Intracellular and Extracellular Calcium Levels

By definition, intracellular Ca^2+^ levels correspond to the concentration of calcium ions in the ER ([Ca^2+^]_ER_), while the extracellular Ca^2+^ levels refer to the concentration of calcium ions in the cytosol ([Ca^2+^]_cytol_). Physiological salt solution (PSS) (140 mM NaCl, 5.9 mM KCl, 1.4 mM MgCl_2_·6H_2_O_2_, 10 mM HEPES, 11.5 mM glucose, 1.2 mM NaH_2_PO_4_, 5 mM NaHCO_3_, and 1.8 mM CaCl_2_, at pH 7.4 with NaOH) was used as the basal reagent. To measure [Ca^2+^]_cytol_ levels, the Ca^2+^ -sensitive Fluo-4 probe was dissolved in PSS. On the contrary, to measure [Ca^2+^]_ER_ levels, the Fluo-4 probe was dissolved in a modified version of PSS, without CaCl_2_. EA.hy926 cells were seeded in 96-well plates overnight when they reached approximately 80% confluence. Next, 1× Fluo-4 was added, and cells were incubated for 30 min at 37 °C in the dark. The cells were rinsed twice with 1× phosphate-buffered saline (PBS), and 50 μL of 1× PBS was added to the wells. Fluorescence intensity was measured for 10 s at 1-s intervals. The cells were then treated with 1× PSS (Control) or DPHC at different concentrations (6, 20, and 60 μM) dissolved in 0.1% bovine serum albumin (BSA). After the treatment, fluorescence was detected for another 50 s at 1-s intervals. To investigate the relationship between DPHC and either AchR or VEGFR2 activation, cells were pretreated for 2 h with 60 μΜ of atropine, an AchR antagonist, and 100 μM of SU5416, a VEGFR2 antagonist. The 1× Fluo-4 fluorescent dye was added, and cells were incubated for another 30 min at 37 °C in the dark. Calcium levels were then measured as described earlier. The box plot data represent the mean value of the AUC calculated from 0 to 60 s. The error bars indicate the maximum and minimum values.

### 4.6. Western Blot Analysis

EA.hy926 cells were treated with different concentrations (0, 6, 20, and 60 μM) of DPHC. After 24 h, cells were washed and harvested with ice-cold PBS and lysed with a lysis buffer on ice for 1 h. Lysates were centrifuged at 12,000 rpm for 20 min, and the protein concentration in the supernatant was evaluated with a BSA protein assay kit (Bio-Rad, Hercules, CA, USA). Next, sodium dodecyl sulfate-polyacrylamide gel electrophoresis (SDS-PAGE) was performed with 10% gel. Proteins were transferred onto a nitrocellulose membrane. Membranes were incubated at 4 °C overnight with the following primary antibodies added separately: anti-β-actin (sc-47778, Santa Cruz Biotechnology, CA, USA; 1:1000), anti-*p*-AKT (sc-377556, Santa Cruz Biotechnology; 1:1000), anti-*p*-eNOS (#9571S, Cell Signaling Technology, Danvers, Massachusetts, USA; 1:1000), and anti-*p*-PI3K (#17366S, Cell Signaling Technology; 1:1000) dissolved in 5% skim milk. Immunoblots were incubated for another 2 h at room temperature with specific secondary antibodies (1:3000). Protein bands were ultimately developed and photographed using the FUSION SOLO Vilber Lourmat system, Paris, France. The Image J 1.50i software (NIH, USA) was used for quantifying the band intensities.

### 4.7. Maintenance and Fluorescence Intensity Assessment in the Tg(flk:EGFP) Transgenic Zebrafish

The zebrafish experiment received approval from the Animal Care and Use Committee of Jeju National University (Approval No. 2017-0001). The *Tg(flk:EGFP)* zebrafish model was used to investigate the behavior of endothelial cells *in vivo*. Fish were housed in 3-L tanks (Aquatic Habitats, Apopka, FL) containing buffered water (pH 7.5) maintained at 28.5 °C. Fertilized eggs were collected from the bottom of the tank in water (temperature 28.5 °C, pH 7.5, dissolved oxygen 7.0, and conductivity 800 μS) containing methylene blue and were placed in Petri dishes after being washed thoroughly with water and transferred to the incubator. For all the experimental procedures, larvae were maintained in 24-well plates containing egg water (reverse osmosis water containing 60 mg sea salt per liter of water, pH 7.5). Larvae were treated with 0, 0.06, 0.2, and 0.6 μM of DPHC at 3 dpf. After six days of treatment, larvae were photographed using a fluorescence microscope at 4× magnification to capture blood vessels in the whole body. Fluorescence intensity signals of the whole body were measured using the Gen5 3.04 software (Bioteck). Fluorescence images were imported into the Image J software to calculate the corrected total object fluorescence (CTOF), according to the following formula:

CTOF = Integrated density − (Area of selected object × mean background fluorescence)

### 4.8. Statistical Analysis

All experiments were conducted in triplicates, and data are shown as mean ± standard deviation. Statistical analysis was performed using the one-way analysis of variance (ANOVA) with Dunnett’s post hoc test with the Prism 5.0 software (GraphPad Software, La Jolla, CA, USA). The following *p*-values were considered statistically significant, and they have been illustrated with asterisks in all figures: * *p* < 0.05, ** *p* < 0.01, and *** *p* < 0.001.

## 5. Conclusions

In this study, we demonstrated the molecular mechanisms underlying the vasodilatory effect of the marine polyphenol, DPHC. DPHC promoted endothelium-dependent vasodilation by modulating calcium concentration through AchR and VEGFR2 activation, thereby triggering NO synthesis through activation of the PI3K/Akt/eNOS pathway. Therefore, DPHC is a potential novel natural compound for vasodilation and the prevention of endothelium-dependent cardiovascular diseases.

## Data Availability

The data sets generated and/or analyzed during the current study are available from the corresponding author on a reasonable request.

## Supplementary Materials

The following are available online at https://www.mdpi.com/1422-0067/22/4/1610/s1.
