# Supplementary figures and images for: Diphlorethohydroxycarmalol Isolated from Ishige okamurae Exerts Vasodilatory Effects via Calcium Signaling and PI3K/Akt/eNOS Pathway
Source: Int J Mol Sci. 2021 Feb 5;22(4):1610. doi: 10.3390/ijms22041610 (PMC7914902; doi:10.3390/ijms22041610)

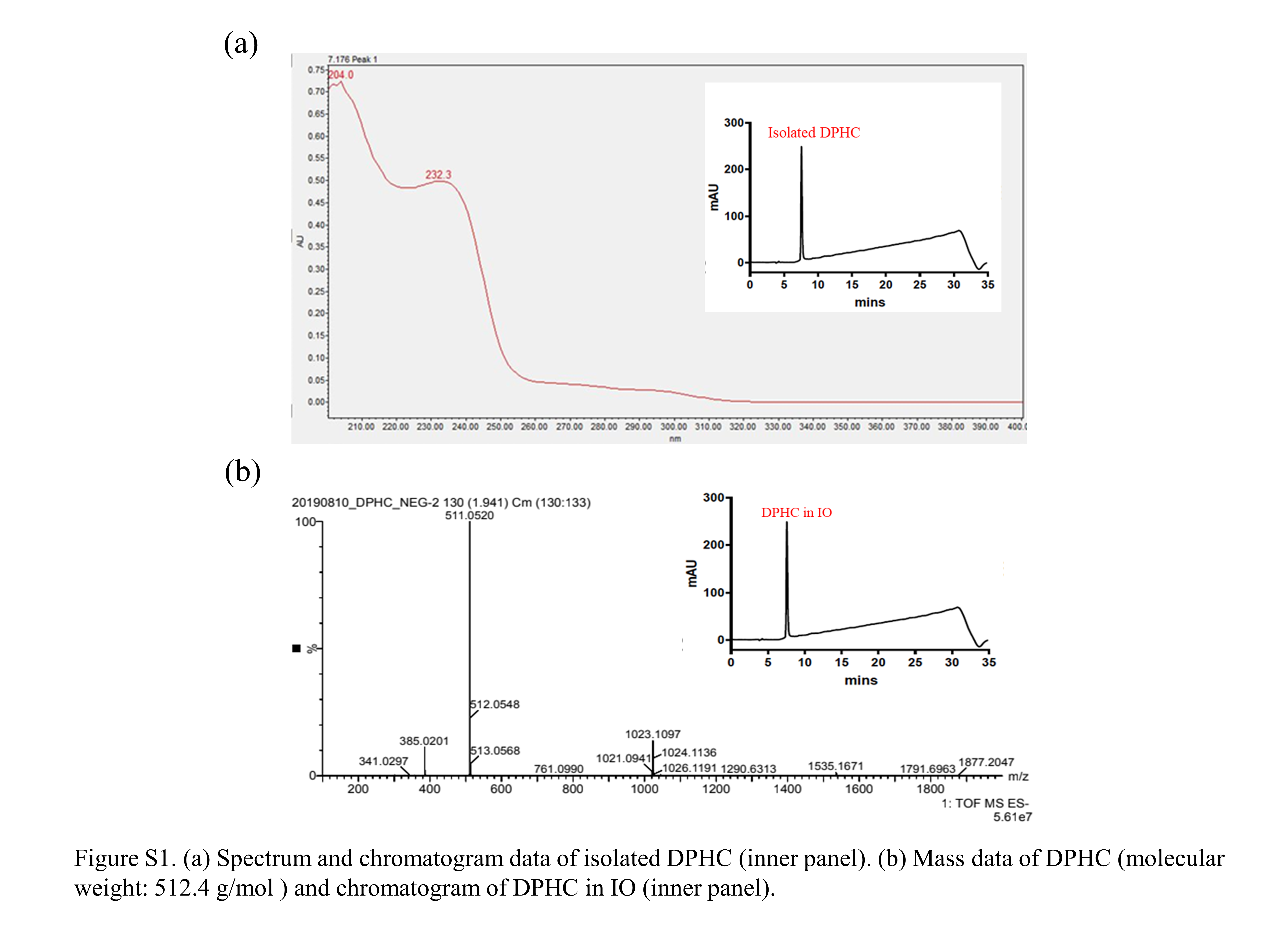

Supplement: Supplementary file 1 [file ijms-22-01610-s001.zip › supplementary data/ijms-1052335 supplementary done.tif]
